# Deletion 22q13.3 syndrome

**DOI:** 10.1186/1750-1172-3-14

**Published:** 2008-05-27

**Authors:** Mary C Phelan

**Affiliations:** 1 Cytogenetics Laboratory, Molecular Pathology Laboratory Network, 250 East Broadway, Maryville, TN 37804, USA

## Abstract

The deletion 22q13.3 syndrome (deletion 22q13 syndrome or Phelan-McDermid syndrome) is a chromosome microdeletion syndrome characterized by neonatal hypotonia, global developmental delay, normal to accelerated growth, absent to severely delayed speech, and minor dysmorphic features. The deletion occurs with equal frequency in males and females and has been reported in mosaic and non-mosaic forms. Due to lack of clinical recognition and often insufficient laboratory testing, the syndrome is under-diagnosed and its true incidence remains unknown. Common physical traits include long eye lashes, large or unusual ears, relatively large hands, dysplastic toenails, full brow, dolicocephaly, full cheeks, bulbous nose, and pointed chin. Behavior is autistic-like with decreased perception of pain and habitual chewing or mouthing. The loss of 22q13.3 can result from simple deletion, translocation, ring chromosome formation and less common structural changes affecting the long arm of chromosome 22, specifically the region containing the *SHANK3 *gene. The diagnosis of deletion 22q13 syndrome should be considered in all cases of hypotonia of unknown etiology and in individuals with absent speech. Although the deletion can sometimes be detected by high resolution chromosome analysis, fluorescence *in situ *hybridization (FISH) or array comparative genomic hybridization (CGH) is recommended for confirmation. Differential diagnosis includes syndromes associated with hypotonia, developmental delay, speech delay and/or autistic-like affect (Prader-Willi, Angelman, Williams, Smith-Magenis, Fragile X, Sotos, FG, trichorhinophalangeal and velocardiofacial syndromes, autism spectrum disorders, cerebral palsy). Genetic counseling is recommended and parental laboratory studies should be considered to identify cryptic rearrangements and detect parental mosaicism. Prenatal diagnosis should be offered for future pregnancies in those families with inherited rearrangements. Individuals with deletion 22q13 should have routine examinations by the primary care physician as well as genetic evaluations with referral to specialists if neurological, gastrointestinal, renal, or other systemic problems are suspected. Affected individuals benefit from early intervention programs, intense occupational and communication therapies, adaptive exercise and sport programs, and other therapies to strengthen their muscles and increase their communication skills. No apparent life-threatening organic abnormalities accompany the diagnosis of deletion 22q13.

## Disease name and synonyms

Deletion 22q13.3 syndrome

Deletion 22q13 syndrome

22q13 deletion syndrome

Phelan-McDermid syndrome

Monosomy 22q13

## Definition and diagnostic criteria

The deletion 22q13 syndrome (or Phelan-McDermid syndrome) is a microdeletion syndrome characterized by severe neonatal hypotonia (>97%) and global developmental delay (>98%), normal to accelerated growth (95%), absent to severely delayed speech (>98%), and minor dysmorphic features. Although the characteristic features are non-specific, the concomitant occurrence of these finding should prompt the clinician to suspect this diagnosis. The diagnosis is based on cytogenetic, molecular cytogenetic, and/or molecular demonstration of loss or disruption of chromosome region 22q13.3 which contains the *SHANK3/ProSAP2 *gene [1-3].

## Epidemiology

The incidence of deletion 22q13 syndrome has not been determined. The deletion occurs with equal frequency in males and females and has been reported in mosaic and non-mosaic forms. The structural abnormalities may be inherited or *de novo*. This syndrome is clearly under-diagnosed at both the laboratory and the clinical levels. Over 30% of individuals with Phelan-McDermid syndrome have required two or more chromosome studies to detect the deletion cytogenetically. Adding FISH (fluorescence *in situ *hybridization) and array CGH (comparative genomic hybridization) to the diagnostic armamentarium have significantly enhanced the detection of this deletion. The analyses of data collected from subtelomeric studies suggest that deletion 22q13 is second to deletion 1p36 as the most common terminal deletion leading to a clinically significant chromosomal disorder [4,5].

## Clinical description

The clinical characteristics of deletion 22q13 syndrome are presented in Table 1. At birth, the features of Phelan-McDermid syndrome are quite subtle. Birth weight is appropriate for gestational age and physical features are often unremarkable. Common physical traits include long eye lashes, large or unusual ears, relatively large hands, dysplastic toenails, full brow, dolicocephaly, full cheeks, bulbous nose, and pointed chin. Hypotonia, feeding problems, and developmental delay are often the early presenting symptoms of deletion 22q13 syndrome. The presence of neonatal hypotonia may be the only indicator that genetic studies are warranted. The Phelan-McDermid syndrome should be considered in all cases of neonatal hypotonia of unknown etiology.

**Table 1:** Clinical characteristics of deletion 22q13 syndrome

***Occurring in >95%***
Severe global developmental delay
Absent/severe speech delay
Hypotonia
Normal/accelerated growth
***Occurring in >75%***
Long eye lashes
Prominent, dysplastic ears
Relatively large, fleshy hands
Hypoplastic/dysplastic toenails
Decreases sensitivity to pain

***Occurring in >50%***
Dolicocephaly
Full brow
Prominent/dysplastic ears
Full/puffy cheeks
Full/puffy eyelids
Pointed chin
Deep set eyes
Ptosis
Decreased perspiration/tendency to overheat
Flat midface
Wide nasal bridge
Bulbous nose
Sacral dimple

***Occurring in >25%***
Cyclic vomiting
Strabismus
Epicanthal folds
Wide spaced teeth/malocclusion
2–3 syndactyly of the toes
Fifth finger clinodactyly
Seizures
Lymphedema
Gastroesophageal reflux
Renal abnormalities

***Occurring in <25%***
Hearing loss (~20%)
Arachnoid cyst (~15%)
Cellulitis (~10%)

***Behavioral features***
Poor eye contact
Stereotypic movements
Decreased socialization
Language impairment
Chewing/mouthing of non-food items (80–90%)
Teeth grinding (about 25%)
Tongue thrusting (about 15%)
Aggressive behavior (10–15%)

Behavioral features of Phelan-McDermid syndrome include poor eye contact, stereotypic movements, decreased socialization, and language impairment consistent with autism spectrum disorders [6-10]. About 3–6% of autistic individuals have a chromosome abnormality identified by standard karyotyping. Deletion 22q13 has been shown to be one of the common chromosome defects associated with autism [4]. The term "syndromic autism" has been suggested for autism accompanied by dysmorphic features and the 22q13 deletion syndrome was cited as an example of a genetic disorder characterized by autistic behavior [11]. Other specific behavioral characteristics include: chewing/mouthing of non-food items, teeth grinding, tongue thrusting and aggressive behavior [10].

## Etiology

The Phelan-McDermid syndrome is a microdeletion syndrome resulting from loss of 22q13 by simple deletion, unbalanced translocation, ring chromosome formation, or other unbalanced structural change [10]. The *SHANK3 *gene maps to 22q13.3 and codes for a structural protein found in the post-synaptic density. SHANK3 functions to connect ion channels and receptors in the post-synaptic membrane to the cytoskeleton and to signal transduction pathways. Haploinsufficiency of the *SHANK3/PROSAP2 *gene is the cause of the major neurological features associated with deletion 22q13 [1,3]. The disruption of the *SHANK3/ProSAP2 *gene by an apparently balanced rearrangement and the alteration of SHANK3 by a frameshift mutation have also been described as mechanisms leading to the language deficits and autistic features of Phelan-McDermid syndrome [2,6].

The size of the deleted region in Phelan-McDermid syndrome ranges from 100 kb to over 9 Mb. The minimum region of overlap is a 90 kb region delineated proximally by cosmid n66C4 and distally by cosmid n94h12 [12]. A recurrent breakpoint within a 15 base pair region of the *SHANK3 *gene has been identified in three individuals with Phelan-McDermid syndrome [13].

## Diagnostic methods

Chromosome analysis at or above the 550-band level will detect most deletions of 22q13 although other, more precise methods are recommended to confirm the deletion. FISH is often used to confirm the presence of the deletion and to rule out the presence of a cryptic translocation. The probe for arylsulfatase A (ARSA) successfully detects the majority of 22q13.3 deletions. Microdeletions distal to ARSA may require FISH analysis using the 22q sub-telomere probe [6,14]. If there is a high degree of clinical suspicion, FISH for ARSA and the 22q sub-telomere should be used simultaneously or sequentially. Using a probe set that targets each of the 41 sub-telomere regions has the advantage of detecting cryptic translocations that will be missed if only distal 22q is targeted. Array-based CGH is more sensitive than FISH and can scan the entire genome for loss or gain of genetic material [7,13,14]. Array CGH is capable of detecting unbalanced rearrangements associated with Phelan-McDermid syndrome by identifying the loss of genetic material from 22q13 while demonstrating the gain of material from a second chromosomal region. While array CGH can identify deletions and unbalanced translocations, standard karyotyping or FISH is required to detect balanced rearrangements in parents or other family members.

## Differential diagnosis

The deletion 22q13 syndrome excludes the diagnoses of chromosomal disorders unrelated to deletion or disruption of the 22q13 region on the long arm of chromosome 22. Most of the disorders included in the differential diagnosis of Phelan-McDermid syndrome are associated with hypotonia, developmental delay, speech delay and/or autistic like affect [14].

### Prader-Willi syndrome

As with Prader-Willi syndrome, neonatal hypotonia can be the first presenting symptom of deletion 22q13. Testing for deletion 22q13 should be considered for any child with a history of neonatal hypotonia of unknown etiology.

### Angelman syndrome

Many individuals with Phelan-McDermid syndrome have carried an initial diagnosis of atypical Angelman syndrome. Features common to Angelman syndrome and deletion 22q13 include global developmental delay, absent speech, ataxic gait, and minor dysmorphic features.

### Autism spectrum disorders

The behavioral characteristics of individuals with Phelan- McDermid syndrome have led to the diagnosis of autism spectrum disorders. In fact, 22q13 has been described as a "hot spot" for these disorders [15]. Although autism or autistic-like behavior is frequently observed in Phelan-McDermid syndrome, laboratory studies to identify deletion 22q13 can distinguish this syndrome from idiopathic autism or other types of syndromic autism [7].

### Cerebral palsy

Cerebral palsy is a non-specific term for a variety of neurological disorders that are typically present at birth and affect motor functions. Neonatal hypotonia, poor coordination, delayed walking and unsteady gait are features that may lead a clinician to misdiagnose a child with Phelan-McDermid syndrome as having cerebral palsy.

### Velocardiofacial syndrome (VCF)/22q11.2 deletion syndrome

Deletion 22q13 has been fortuitously diagnosed by FISH in a number of individuals initially tested for VCF. Many of the commercially available FISH assays for VCF use ARSA as the control probe. ARSA maps to 22q13 and therefore detects deletions of this region. Features common to VCF and Phelan-McDermid syndrome include hypotonia, broad nasal root, epicanthal folds, narrow palpebral fissures, renal abnormalities, speech delay, and developmental delay.

### Williams syndrome

Newborns and young children with Williams syndrome may have hypotonia, puffy eyelids, full cheeks, and global delay, similar to individuals with Phelan-McDermid syndrome. The diagnosis of Williams syndrome can be confirmed or excluded by FISH studies targeting the elastin (*ELN*) gene at 7q11.2, just as appropriate laboratory studies can detect deletion 22q13.

### Trichorhinophalangeal syndrome (TRP)

Features shared by TRP and Phelan-McDermid syndrome include hypotonia, bulbous nose, large or prominent ears, deep set eyes, hypoplastic toenails, and developmental delay. Microdeletions of 8q24 are associated with TRP.

### Smith-Magenis syndrome (SMS)

Hypotonia, speech deficit, delayed motor skills, developmental delay, flat midface, and decreased sensitivity to pain are features common to Smith-Magenis syndrome and Phelan-McDermid syndrome. Deletion 17p11.2 which can be detected by chromosome analysis, FISH, or CGH-arrays confirms the diagnosis of SMS.

### Fragile X syndrome

Males with deletion 22q13 who have hypotonia, speech delay, autistic-like behavior and certain physical features including large hands, large ears, and tall stature have been misdiagnosed with fragile X syndrome. The diagnosis of fragile syndrome is confirmed by molecular analysis demonstrating the CGG repeat in the *FMR1 *gene.

### FG syndrome

Features common to FG syndrome and Phelan-McDermid syndrome are hypotonia, delayed speech, developmental delay, and autistic-like behavior.

### Sotos syndrome

Sotos syndrome is also known as cerebral gigantism and is characterized by general overgrowth. As in Phelan-McDermid syndrome, normal birth weight and length, neonatal hypotonia, poor feeding, motor delay, and developmental may be seen in Sotos syndrome. Similar features include dolicocephaly, pointed chin, and large hands.

## Genetic counseling

Targeted laboratory studies are warranted in parents of individuals with deletion 22q13. The possibility of parental mosaicism for a simple deletion or structural rearrangement that could result in an affected child should be considered in the laboratory analysis. About 80% of individuals with Phelan-McDermid syndrome have *de novo*, simple deletions of 22q13 [9]. As in other terminal deletion syndromes, the deletion occurs preferentially on the paternally-derived chromosome 22 [1,3]. Cryptic and half-cryptic rearrangements have also been identified in Phelan-McDermid syndrome [10].

About 20% of cases of deletion 22q13 result from unbalanced structural rearrangements [1]. Structural abnormalities most commonly associated with deletion 22q13 syndrome are unbalanced translocations and ring chromosomes. The identification of a balanced translocation involving chromosome 22 in one parent significantly increases the risk of recurrence in future pregnancies. Several families have experienced multiple cases of deletion 22q13 secondary to familial chromosome translocations. Mother to son transmission of an insertional translocation has resulted in deletion 22q13 in the son [16]. Recombination of a parental pericentric inversion has also led to a child with deletion 22q13 [17-19]

Transmission of ring chromosome 22 from parent to offspring has been described. In familial cases of ring 22, the ring chromosome typically results from an end-to-end fusion of the long and short arms of chromosome 22, with no apparent loss of genetic material. However, at least one case of Phelan-McDermid syndrome is known to result from the transmission of a ring chromosome from a mosaic mother to the affected child (Phelan, personal communication).

## Antenatal diagnosis

Deletion of 22q13 can be detected in amniotic fluid, chorionic villi, and percutaneous umbilical blood samples. As in postnatal studies, the deletion is subtle and is often difficult to detect. If deletion of 22q13 is suspected by banded chromosome, FISH using a probe specific for 22q13 or CGH micro-array analysis should be performed to confirm this finding.

Antenatal diagnosis should be offered to parents who carry structural rearrangements that predispose to deletion of 22q13. Simple deletion of 22q13 has not recurred in offspring of parents with normal karyotypes. However, in one case, a phenotypically normal parent with low level mosaicism (6%) for deletion 22q13 gave birth to affected siblings (Phelan, unpublished). In the absence of extensive laboratory analysis, low level parental mosaicism may not be suspected until the birth of a second affected child.

## Management

Individuals with Phelan-McDermid syndrome should receive routine medical care from their primary physician. A clinical geneticist should be involved following the initial diagnosis. Periodic genetic evaluations are warranted to update parents as more information on this syndrome becomes available. For symptoms affecting particular organ systems, specialists should be consulted [10]. A neurologist should be consulted for hypotonia and seizures. For cyclic vomiting, a neurosurgical consult is appropriate to address issues related to increased intra-cranial pressure. Arachnoid cysts occur more frequently in children with deletion 22q13 than in the general population. Individuals with Phelan-McDermid syndrome should have baseline brain imaging studies upon initial diagnosis and should be re-evaluated if clinical symptoms of increased cranial pressure occur. Other neurological problems include ventriculomegaly and reduced myelination.

Recurrent ear infections, along with the lack of expressive language, may lead to concerns about hearing deficits. Typanostomy tubes may be required to treat recurrent ear infections. A specialist experienced in testing children with severe developmental delay and communication deficits should evaluate hearing in children with deletion 22q13 syndrome.

Gastroesophageal reflux is generally controlled by medication. In persistent cases, surgery may be required. Recurrent vomiting may require intravenous fluids to prevent dehydration. Because renal malformations are observed in over 25% of individuals with Phelan-McDermid, a renal ultrasound should be obtained upon initial diagnosis.

Cardiac, respiratory, immunologic, and other medical issues should be treated in the same manner as they would be handled in a typical child.

An occupational therapist, physical therapist and nutrition specialist should evaluate feeding problems and early motor delays related to hypotonia. A child development specialist should assess children for autism or autistic-like features. Medication to reduce hyperactivity and self-stimulatory behavior is helpful in certain children.

Children with Phelan-McDermid syndrome often have more advanced perceptive language than expressive language. Therapies should be directed at improving verbal and nonverbal communication. Sign language, computer touch screens, voice based systems, and picture exchange systems have been used to increase communication skills. Adaptive sports, music therapy, and sensory integration increase the child's awareness and often improve their desire to communicate. Children with deletion 22q13 greatly benefit from aggressive infant stimulation programs and individual education plans to improve their motor skills and communication skills.

## Prognosis

Individuals with deletion 22q13 typically have no life-threatening organic malformations. The number of older adults with this syndrome is small, therefore the potential for developing life-threatening conditions with age is unknown. Deletion 22q13 is a life long disability. Affected individuals may function well in a group home setting. It is unlikely that individuals could function independently without supervision of parents or other caretakers.

## Unresolved questions

• Frameshift mutation of *SHANK3 *has been associated with the neurological deficits in Phelan-McDermid syndrome. Gene sequencing studies are needed to identify additional mutations of *SHANK3 *and their role in this disorder.

• Although a plausible role for *SHANK3 *has been determined, no additional genetic and/or environmental factors important in the pathogenesis of this syndrome have been identified.

• Previous studies have not shown a strong correlation between phenotype and deletion size. Larger series of patients are required to resolve this question.

## Competing interests

The author declares that they have no competing interests.
